# A case report of anal canal cancer with pagetoid spread requiring differential diagnosis

**DOI:** 10.1016/j.ijscr.2020.09.048

**Published:** 2020-09-10

**Authors:** Ryohei Yukimoto, Shiki Fujino, Norikatsu Miyoshi, Takayuki Ogino, Hidekazu Takahashi, Mamoru Uemura, Atsushi Tanemura, Chu Matsuda, Hirofumi Yamamoto, Tsunekazu Mizushima, Yuichiro Doki, Hidetoshi Eguchi

**Affiliations:** a Osaka University, Graduate School of Medicine, Department of Gastroenterological Surgery, Osaka, Japan; b Department of Dermatology, Osaka University Graduate School of Medicine, Osaka, Japan

**Keywords:** APR, abdominoperineal resection, CT, computed tomography, PS, pagetoid spread, Paget’s disease, Diagnosis, Anorectal cancer, Case report

## Abstract

**Introduction:**

Paget’s disease is an intraepithelial invasion by a malignant tumour and is characterised by erythema and inflammation. It can manifest as mammary or extramammary Paget’s disease (EMPD), with the latter often developing in the perianal area. Anorectal cancer can cause transepithelial invasion into the epidermis, resulting in an appearance similar to that of EMPD. This is called pagetoid spread (PS), which is completely different from EMPD. These two conditions are difficult to differentiate because of the nature of intricacy and requirements of histopathology.

**Presentation of case:**

We present a case in which differential diagnosis between these two conditions was not possible during the preoperative examination, resulting in difficulties in treatment. The patient was a 70-year-old woman who experienced pain in the anus since the previous month and presented with red, flat and elevated lesions.

**Discussion:**

Treatment for dermatitis was ineffective, and endoscopic examination did not indicate rectal or anal cancer. However, immunohistochemical examination of the biopsy specimen suggested PS. Thus, two-stage operation was planned. Transanal surgery was performed to confirm the diagnosis of PS and intersphincteric resection was allowed as a radical surgery.

**Conclusion:**

Thus, when differentiation between EMPD and PS is intricacy, two-stage operation is useful in selecting an appropriate radical surgery method, leading to preservation of anal function.

## Highlights

• Pain in the anus or presented with red, flat, elevated lesions in the anus needs to suspecte anal canal cancer.
• To differentiate between extramammary Paget’s disease (EMPD) and Anorectal cancer with pagetoid spread (PS) is difficult.
• A two-stage operation, a local excision followed by pathological diagnosis and an additional excision,can be useful in PS.

## 1 Introduction

Perianal Paget’s disease and pagetoid spread (PS) associated with anorectal cancer both present with anal inflammation and erythema. Although these two diseases are clinically similar, the treatment method and prognoses are notably different [1]. To differentiate between these two diseases, colorectal endoscopy, computed tomography (CT), magnetic resonance imaging (MRI) or immunohistochemical examination are often used. However, definitive diagnosis and treatment can be difficult to achieve [2]. Here, we report a planned two-stage operation case in which transanal surgery was used to achieve a definitive diagnosis and select an appropriate treatment method to conserve anal function. The work has been reported in line with the SCARE criteria [3].

## 2 Presentation of case

**Fig. 1:**
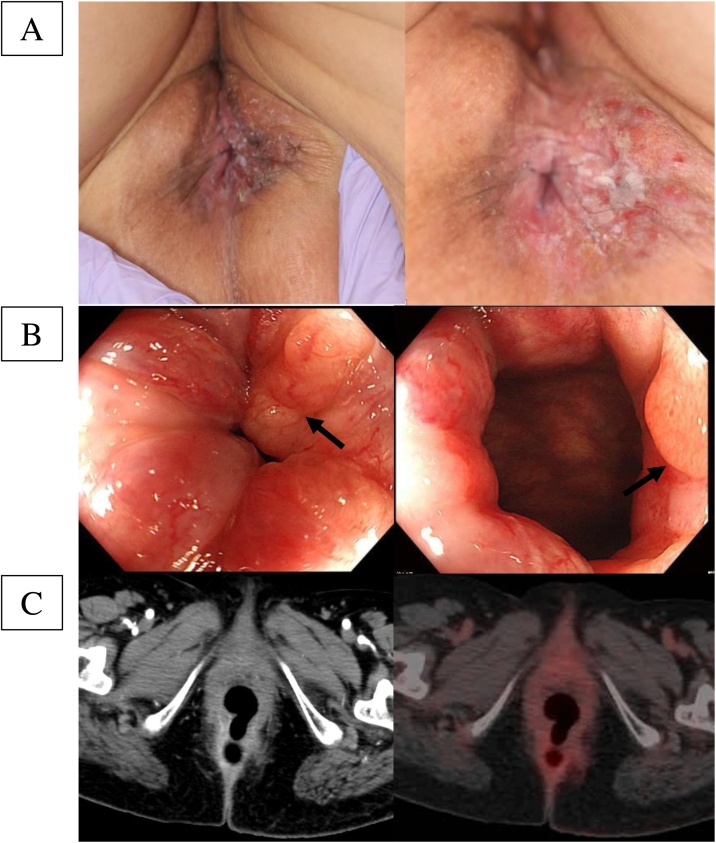
Preoperative examination. a. Visual inspection revealed flat, elevated lesions and surrounding inflammation on the left side of the anus. b. Colonoscopy revealed an elevated lesion continuing from the anal skin lesion in the 3 o’clock direction of the anal canal, going toward, but not reaching, the pectinate line. c. There was not any abnormal accumulation in the anus or rectum through a CT scan and 18F-FDG PET/CT scans.

The patient was a 70-year-old woman who had developed red and flat elevated lesions, with surrounding inflammation on the left side of the anus that persisted for one year before treatment (Fig. 1a). She began experiencing pain in the anus a month prior to treatment, and subsequent treatment for dermatitis was ineffective.

Colorectal endoscopy confirmed an elevated lesion continuing from the anal skin lesion in the 3 o’clock direction toward, but not reaching, the pectinate line (Fig. 1b). There was no abnormality in the rectal mucous membrane. We could not find any visible indications of primary colorectal cancer and lymph node metastasis through a preoperative CT scan, MRI and ^18^F-FDG PET/CT scans (Fig. 1c). Moreover, there was no metastasis in groin lymph node on preoperative examinations.

**Fig. 2:**
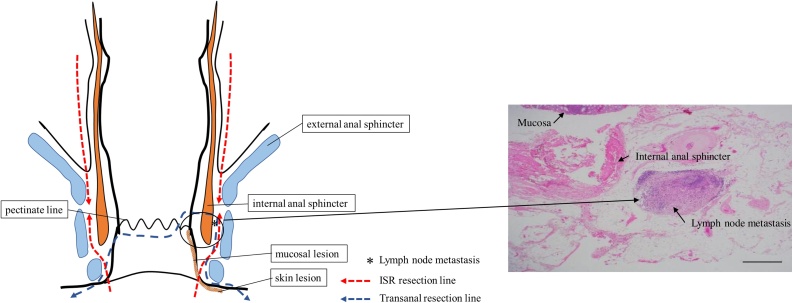
The schema showed resection line and lymph node metastasis (*). Photomicrograph of resected specimen showed lymph node metastasis. Blue line transanal resection line, and red line is resection line of ISR.

Pathological examination of the skin biopsy specimen confirmed multiple Paget cells. On immunohistochemical testing, the samples stained positive for cytokeratin-7 (CK7) and cytokeratin-20 (CK20), but not for gross cystic disease fluid protein-15 (GCDFP15; Fig. 2). These results strongly suggested PS. However, since the Colonoscopy and CT did not confirm a primary neoplastic lesion, we could not confirm the diagnosis. For procedure, we performed a transanal resection with local excision of the skin and full-thickness skin grafting.

Our strategy was to precede transanal local resection with local excision of the skin and full-thickness skin grafting for diagnostic purpose and to determine the need for radical surgery with a pathological diagnosis.

**Fig. 3:**
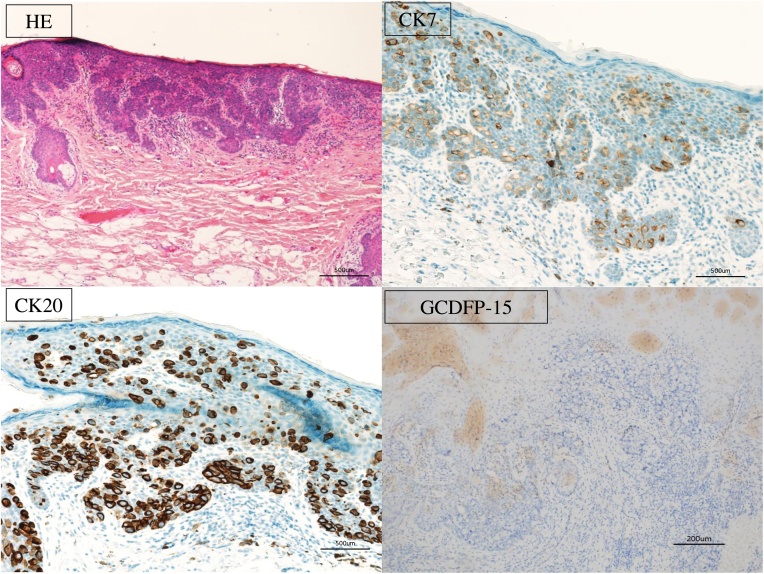
The figures showed HE staining and immunohistochemical staining of the skin biopsy. They revealed that the signal for GCDFP15 was negative, whereas those for CK7 and CK20 were positive (×10, ×100).

During surgery, concordant with the endoscopy result, we observed that the mucosal lesion continued for approximately 3 cm in the 3 o’ clock direction from the skin lesion up the anal canal but did not reach the pectinate line. Therefore, we made en-bloc resection of perianal lesion of the cutaneous disease to secure a margin of approximately 1 cm from the mucosal lesion. Thus, the external anal sphincter was preserved (Fig. 3). Pathological examination of resected specimens revealed anal adenocarcinoma with lymph node metastasis. Immunostaining of the tumor showed positive signals for CK7 and CK20. Hence, additional surgical resection was deemed necessary, and laparoscopic total intersphincteric resection (ISR) and a temporary ileostomy were performed. Pathological examination of the additional resection specimen did not reveal any malignancy. The patient was discharged thirteen days post-operatively without complication. During the final postoperative pathological examination, the tumour was categorised as P, Type 2, 20 × 13 mm, tub2, pT2, INFb, ly1, v1, Pn1a, pN1.

The patient is currently undergoing a postoperative adjuvant chemotherapy regimen (FOLFOX), with no recurrence for 17 months.

## 3 Discussion

For perianal inflammation and erythema that do not respond to typical dermatitis treatment, EMPD and PS should be considered strong candidates for the differential diagnosis, for which diagnostic imaging and immunohistochemical examinations should be used [4]. Specifically, if PS is suspected, it is essential to examine for malignancy in adjacent organs by conducting a lower endoscopic examination. However, as observed in this case, if malignancy is not confirmed despite the spread of the lesion to the anal mucous membrane and PS is suspected based on the immunohistochemical examination, the treatment modality can be difficult to determine. In such scenarios, with respect to anal function, procedure such as transanal surgery can be performed for the mucosal lesion. The specimen should subsequently be analysed to make a definitive diagnosis and develop a treatment strategy.

It has been reported that approximately 20% of perianal skin lesions are malignant [4]. Both primary Paget’s disease of the skin and PS are clinically characterised by inflammation and erythema. In the latter case, cancer in an organ adjacent to the skin spreads and develops conditions similar to those of intra-epidermal carcinoma. However, both diseases have their own unique primary lesions, and their treatment methods and prognoses are completely different. We summarised 10 cases of PS (Table 1), and our case was the case to perform ISR for anal adenocarcinoma with PS. Eight patients with PS who had associated anorectal cancer underwent abdominoperineal resection (APR). Additionally, rectal excision often results in decreased postoperative quality of life (micturition problems, gastrointestinal tract symptoms, defecation problems, stoma-related problems and male and female sexual problems) [5]. In order to avoid overtreatments, it is vital to clearly differentiate between Paget’s disease and PS.

**Table 1:** Summary of reports on pagetoid spread (PS) cases, preoperative diagnoses and treatment methods.

Author	Year of report	Chief complaint	Preoperative endoscopic examination	Preoperative endoscopic examination biopsy	Treatment
Tjandra et al. [9]	1988	Pruritus	Rectal lesion	Adenocarcinoma	APR
Lertprasertsuke et al. [10]	1991	None	Tumour	Adenocarcinoma	APR
Goldman et al. [11]	1992	Pruritus	Hard mass in the upper anal canal	Adenocarcinoma	APR
Goldman et al. [11]	1992	Pruritus	Tumour	Adenocarcinoma	Radiation + APR
Kubota et al. [12]	1998	Anal bleeding	Tumour	Adenoma	APR
Suenaga et al. [13]	2006	None	Tumour	Adenocarcinoma	Wide local excision
Ishida et al. [14]	2013	Anal fistula	Tumour	Adenocarcinoma	APR
Shimizu et al. [15]	2017	Anal pain	No significant finding	No	APR
Matsubara et al. [16]	2017	Warts	No significant finding	No	APR
Yamamura et al. [2]	2018	None	Tumour	Adenocarcinoma	Chemotherapy
This case	2019	Anal pain	No significant finding	No	ISR

ISR: intersphincteric resection; APR: abdominoperineal resection.

In a few cases out of the 10 that we summarised, when differentiating between EMPD and PS, either endoscopic examination or diagnostic imaging revealed the possibility of anal/rectal cancer, which in turn led to preoperative pathological diagnosis. Eight out of 10 cases had preoperative malignant findings (Table 1). There were two cases with no malignant finding in the preoperative examination, such as our case, and APR has also been selected for these two cases. Notably, some reports state that it is not easy to distinguish between secondary and primary EMPD based on clinical and histological findings, particularly if primary EMPD invades the epidermis or if an underlying visceral carcinoma is not apparent [2].

There are several reports on the usefulness of immunohistochemical staining for differentiation. GCDFP-15, which is expressed in apocrine and eccrine sweat glands, and CK7 and CK20, which are highly expressed in the gastrointestinal mucosal epithelium of rectal cancer, are widely used as markers for differentiation. Generally, GCDFP-15 staining should be positive and CK20 staining should be negative for Paget’s disease, whereas the inverse is true for PS [6]. Among the available reports of PS cases, we summarised eight cases for which the results of immunohistochemical staining were reported (Table 2). In all eight cases, GCDFP-15 staining was negative and CK20 staining was positive. The findings of the present case were the same, confirming that immunohistochemical staining has good specificity in this regard. However, there have also been a few cases of adenoma and perianal EMPD with positive CK20 and negative GCDFP-15 staining [6,7]. Additionally, a report on the efficacy of CK7 and CK20 immunohistochemical staining showed that among 45 cases of primary EMPD and 16 cases of secondary EMPD (primary lesions in the anorectal area, bladder and prostate), CK20 was positive in 22% of the primary EMPD cases and 50% of the secondary EMPD cases. This result is different from those of other reports, suggesting that immunohistochemical staining, as a method to differentiate between Paget’s disease and PS, is not sufficient on its own [8].

**Table 2:** Summary of the results of immunohistochemical staining for CK20 and GCDFP-15 in pagetoid spread (PS) cases.

Case	Year of report	Sex	Age	CK7	CK20	GCDFP-15
Goldblum et al. [7]	1988	Male	66	+	+	–
Goldblum et al. [7]	1988	Female	74	+	+	–
Goldblum et al. [7]	1988	Female	81	+	+	–
Goldblum et al. [7]	1988	Female	89	+	+	–
Liao et al. [1]	2014	Male	78	+	+	–
Shimizu et al. [15]	2017	Male	81	N.A.	+	–
Matsubara et al. [16]	2017	Female	81	+	+	–
Yamamura et al. [2]	2018	Male	76	+	+	–

In cases that are difficult to diagnose, a two-stage operation, a local excision followed by pathological diagnosis and an additional excision, is considered feasible and useful. In the present case, diagnostic imaging did not suggest PS, whereas immunohistochemistry did, making the differential diagnosis difficult. Thus, we performed enbloc resection of the skin and mucosal lesion to make a definitive diagnosis based on the biopsy specimen. Definitive diagnosis based on analysis of the resected specimen can sometimes lead to a requirement for a more invasive surgery; two additional surgeries were indicated in this case. We opted to perform an ISR and were able to administer radical treatment. In the future, we need to examine whether the present treatment strategy is effective for cases in which differentiation between EMPD and PS is difficult.

## 4 Conclusion

The treatment methods and prognoses for Paget’s disease and PS are notably different, making it extremely important to clearly differentiate between the two conditions. We reported a two-stage operation case in which a transanal resection was performed for diagnosis followed by a radical ISR in a case which was difficult to diagnose EMPD or PS.

## Funding

The authors declare that there are no sources.

## Ethical approval

IRB number: 15144-6 Osaka university.

## Consent

Written informed consent was obtained from the patient for publication of this case report and accompanying images. A copy of the written consent is available for review by the Editor-in-Chief of this journal on request.

## Author contribution

S.F. and N.M. conceptualized the project, designed and performed the experiments, interpreted the results, and co-wrote the manuscript. N.M. supervised the experimental design and interpreted the results. N.M., M.O., and M.Y. performed the surgeries and prepared the culture samples. N.M., T.M., Y.D., and M.M. analysed data or participated in discussions of the results.

## Registration of research studies

1. Name of the registry: Research registry.
2. Unique identifying number or registration ID: Researchregistry5806.
3. Hyperlink to your specific registration (must be publicly accessible and will be checked): https://www.researchregistry.com/browse-the-registry#home/registrationdetails/5f0e8b4f136e4900154e8811/.

## Guarantor

Shiki Fujino is guarantors for this article.

Shiki Fujino: sfujino@gesurg.med.osaka-u.ac.jp.

## Provenance and peer review

Not commissioned, externally peer-reviewed.

## Declaration of Competing Interest

The authors report no declarations of interest.
